# Rezidivierende* Serratia-marcescens*-Bakteriämie: Wer suchet, der findet

**DOI:** 10.1007/s00108-023-01508-y

**Published:** 2023-05-03

**Authors:** Micha Banz, Nedim Memisevic, Mahmoud Diab, Amer Malouhi, Stefan Hagel

**Affiliations:** 1 Institut für Infektionsmedizin und Krankenhaushygiene, Universitätsklinikum Jena, Friedrich-Schiller-Universität Jena, Am Klinikum 1, 07747 Jena, Deutschland; 2 Klinik für Innere Medizin I, Kardiologie, Angiologie und internistische Intensivmedizin, Universitätsklinikum Jena, Friedrich-Schiller-Universität Jena, Jena, Deutschland; 3 Klinik für Herz- und Thoraxchirurgie, Universitätsklinikum Jena, Friedrich-Schiller-Universität Jena, Jena, Deutschland; 4 Institut für Diagnostische und Interventionelle Radiologie, Universitätsklinikum Jena, Friedrich-Schiller-Universität Jena, Jena, Deutschland

**Keywords:** Blutstrominfektion, Spondylodiszitis, Sondenextraktion, CIED-Endokarditis, Kardiale elektronische Implantate, Bloodstream infections, Spondylodiscitis, Lead extraction, CIED-associated endocarditis, Cardiac implantable electronic devices

## Abstract

Ein 79-jähriger Patient wird aufgrund einer rezidivierenden *Serratia-marcescens-*Bakteriämie stationär behandelt. Es konnten eine Infektion der Elektroden des implantierbaren Kardioverter-Defibrillator (ICD) mit septischen pulmonalen Embolien und eine Spondylodiszitis diagnostiziert werden. Zusätzlich zur Antibiotikatherapie erfolgte die komplette Extraktion des ICD-Systems. Bei Patienten mit kardialen elektronischen Implantaten (CIED) und einer nicht hinreichend erklärbaren bzw. rezidivierend auftretenden Bakteriämie, unabhängig davon, um welchen Erreger es sich handelt, muss immer eine CIED-assoziierte Infektion ausgeschlossen werden.

## Abstract (en)

A 79-year-old patient was hospitalized due to recurrent *Serratia marcescens* bacteremia. An implantable cardioverter-defibrillator (ICD) electrode infection with septic pulmonary emboli and vertebral osteomyelitis were diagnosed. In addition to antibiotic therapy, the ICD system was completely extracted. In patients with cardiac implantable electronic devices (CIED) and bacteremia that cannot be adequately explained or recurs, regardless of the pathogen involved, a CIED-associated infection always needs to be ruled out.

## Anamnese

Wir berichten über einen 79-jährigen männlichen Patienten, der sich mit Fieber, Schüttelfrost, Nachtschweiß (2- bis 3‑mal/Nacht Wechsel der Kleidung nötig) sowie Rücken- und Schulterschmerzen in der Notaufnahme vorstellte. Die Beschwerden würden seit einigen Tagen bestehen, einhergehend mit einer zunehmenden Minderung des Allgemeinzustands. An relevanten Vordiagnosen sind u. a. eine ischämische Kardiomyopathie mit einer hochgradig reduzierten Pumpfunktion, eine ICD-Implantation zur Primärprophylaxe vor 1,5 Jahren, ein Diabetes mellitus Typ 2 sowie ein Harnblasen- und Prostatakarzinom bekannt.

## Befund

Bei Vorstellung in der Notaufnahme hatte der Patient Fieber, ein erhöhtes CRP (271 mg/l) sowie ein erhöhtes Prokalzitonin (4,08 ng/ml). Weiterhin imponierte ein akut-auf-chronisches Nierenversagen. In der körperlichen Untersuchung zeigte sich vor allem ein Klopfschmerz im Bereich der HWS und BWS. Die Atemfrequenz betrug 16/min, die Sauerstoffsättigung 98 % unter Raumluft. Zur Fokussuche erfolgte eine sonographische Untersuchung des Abdomens, welche bis auf einen Zustand nach Cholezystektomie unauffällig war. In der Röntgenthoraxuntersuchung wurde bei fleckförmigen unscharfen Verdichtungen zunächst der Verdacht auf eine Pneumonie gestellt. Es erfolgte nach Abnahme von Blutkulturen die Einleitung einer empirischen Antibiotikatherapie mit Piperacillin/Tazobactam und Azithromycin.

## Diagnose

*S.-marcescens*-ICD-Elektroden-Infektion mit septischen pulmonalen Embolien und Spondylodiszitis (HWK 4/5)

## Therapie und Verlauf

**Figure 1:**
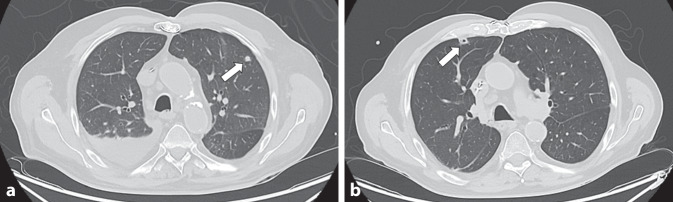

Der Patient wurde auf die Normalstation aufgenommen und die Therapie zunächst fortgeführt. In allen abgenommenen Blutkulturen konnte *Serratia marcescens* nachgewiesen werden, sodass die Therapie mit Azithromycin beendet wurde. Auffällig war, dass dies bereits die dritte Episode einer Bakteriämie mit *S. marcescens* innerhalb der vergangenen 10 Wochen darstellte. Der erste Nachweis erfolgte im Rahmen eines stationären Krankenhausaufenthalts aufgrund einer Harnwegsinfektion mit Nachweis von *S. marcescens *sowohl in der Urin- als auch in der Blutkultur (Episode Nr. 1). Aufgrund eines nur zögerlich abfallenden CRP erfolgte damals eine insgesamt 14-tägige Therapie mit Meropenem bzw. Piperacillin/Tazobactam. Ein weiterer Infektfokus konnte in einer Computertomographie des Thorax und Abdomens ausgeschlossen werden. Die Kontrollblutkulturen waren unauffällig und der Patient wurde nach zweiwöchigem Aufenthalt entlassen. Nur vier Tage nach Entlassung stellte sich der Patient erneut mit Fieber und Schüttelfrost in der Notaufnahme vor. In den Blutkulturen konnte erneut *S. marcescens *kultiviert werden (Episode Nr. 2). Im CT des Thorax zeigten sich im rechten Unterlappen ein neues Infiltrat sowie bipulmonale, herdförmige Konsolidierungen mit spikulierten Ausläufern (Abb. 1). In der zur weiteren Abklärung durchgeführten bronchoalveolären Lavage konnten *Klebsiella oxytoca* und *S. marcescens* in einer Gesamtkeimzahl von 100.000 Keimen/ml kultiviert werden. Unter der Verdachtsdiagnose einer nosokomialen Pneumonie erfolgte eine 9‑tägige Antibiotikatherapie initial mit Meropenem, gefolgt von Ceftriaxon. Nach 10 Tagen wurde der Patient entlassen. Sechs Wochen nach diesem zweiten Aufenthalt erfolgte die aktuelle Aufnahme des Patienten mit der dritten Episode einer *S.-marcescens-*Bakteriämie. Im Vergleich zum CT des Thorax des Voraufenthalts zeigten sich neue Konsolidierungen, während sich die bereits bekannten als größenregedient, teils einschmelzend präsentierten. Es wurde der Verdacht auf ein septisch-embolisches Geschehen geäußert (Abb. 1). Zur weiteren Abklärung erfolgten eine transösophageale Echokardiographie (TEE) und eine Fluordesoxyglukose-Positronenemissionstomographie-Computertomographie (FDG-PET-CT). In der TEE zeigte sich an der ICD-Sonde kurz vor dem Durchtritt durch den Trikuspidalanulus eine längliche flottierende Struktur (ca. 13 × 5 mm). In der FDG-PET-CT zeigte sich eine Spondylodiszitis im Bereich HWK 4/5, ohne Hinweise für eine Endokarditis oder Infektionen des vor zwei Jahren implantierten ICD-Systems. Zudem zeigte sich ein gering gesteigerter Stoffwechsel der multiplen bipulmonalen Verdichtungen. Aufgrund der Verdachtsdiagnose einer ICD-Sonden-Endokarditis mit *S. marcescens *erfolgte im weiteren Verlauf die komplette Extraktion des ICD-Systems (Aggregat inklusive der Sonden). In den intraoperativ abgenommenen mikrobiologischen Kulturen der ICD-Sonde wurde *S. marcescens* kultiviert, sodass die Diagnose bestätigt werden konnte. Die Therapie wurde auf Ciprofloxacin oralisiert und eine operative Versorgung der HWK mittels Osteosynthese durchgeführt. Von einer erneuten ICD-Implantation wurde abgesehen. Retrospektiv kann vermutet werden, dass im Rahmen der ersten Episode der *S.-marcescens*-Bakteriämie eine sekundäre Infektion der ICD-Sonden erfolgte. Nachfolgend kam es, ausgehend von den ICD-Sonden, zu einer hämatogenen Streuung der Erreger mit der Folge von septischen pulmonalen Embolien und einer Spondylodiszitis.

## Diskussion

Infektionen gehören zu den schwerwiegendsten Komplikationen der Therapie mit kardialen elektronischen Implantaten („cardiac implantable electronic devices“ [CIED]). Das Risiko für eine CIED-Infektion liegt bei 0,6–3,4 % im ersten Jahr und steigt mit zunehmender Komplexität der implantierten Aggregate an [1]. Belastbare Daten zur Häufigkeit von CIED-assoziierten Infektionen in Deutschland liegen nicht vor. Aufgrund der Tatsache, dass in Deutschland so viele Herzschrittmacher und ICD implantiert werden wie in keinem anderen europäischen Land, ist jedoch zu vermuten, dass die Anzahl CIED-assoziierter Infektionen entsprechend hoch ist [2]. Häufigste Erreger einer CIED-assoziierten Infektion sind Koagulase-negative Staphylokokken (42–69 %) und *S. aureus* (14–29 %), das Risiko einer CIED-Infektion aufgrund gramnegativer Erreger ist deutlich niedriger (< 10 %; [1]). Bei den gramnegativen Bakterien sind insbesondere *Serratia *spp. und* Pseudomonas aeruginosa* mit einer hohen Rate an CIED-Infektionen assoziiert. In einer retrospektiven Studie der Duke University bei 132 Patienten mit CIED und Bakteriämie mit einem gramnegativen Erreger war die Prävalenz von CIED-Infektionen besonders hoch mit *P. aeruginosa* (54 %; 7 von 13 Patienten) und *S. marcescens* (47 %; 7 von 15 Patienten), während die übrigen gramnegativen Erreger zusammen eine moderate Prävalenz von 7,7 % (8 von 104 Patienten) aufwiesen [3]. Aufgrund dessen sollten CIED-Träger, die eine nicht hinreichend erklärbare bzw. rezidivierende Blutstrominfektion mit *Serratia* spp. oder *P. aeruginosa* haben, besonders auf das Vorliegen einer CIED-assoziierten Infektion hin untersucht werden.

Die definitive Therapie einer CIED-assoziierten Infektion besteht in einer erregerspezifischen Antibiotikatherapie und der kompletten Entfernung des gesamten Systems [1]. Eine alleinige Antibiotikatherapie ohne Systemextraktion geht mit einem Rezidivrisiko von > 50 % einher, wie bei unserem Patienten beobachtet werden konnte. Im vergangenen Jahr wurde erstmals eine gemeinsame Empfehlung der Deutschen Gesellschaft für Kardiologie (DGK) und der Deutschen Gesellschaft für Thorax‑, Herz- und Gefäßchirurgie (DGTHG) zur Explantation von kardialen Devices und Extraktion von Sonden veröffentlicht [4]. Neben Hinweisen zur technischen Durchführung werden Empfehlungen zur Extraktion von infizierten Sonden/Devices gegeben. Entsprechend internationalen Leitlinien wird auch in den deutschen Empfehlungen eine komplette Systemextraktion (Aggregat + Sonden) empfohlen bei

- Vorliegen einer manifesten Tascheninfektion, Taschenerosion sowie einer Taschenperforation (Klasse-I-Indikation = ist indiziert),
- Elektroden- oder Herzklappeninfektion (Endoplastitis/Endokarditis) mit/ohne Tascheninfektion (Klasse-I-Indikation = ist indiziert).

Eine vollständige Entfernung eines infizierten CIED-Systems wird so zeitnah wie möglich, spätestens jedoch innerhalb von 3 Tagen nach Indikationsstellung empfohlen. Dabei sind explizit alle bereits stillgelegten bzw. früher belassenen Sonden mit zu entfernen. Eine ausschließlich lokalchirurgische Behandlung einer Tascheninfektion oder Taschenperforation ohne komplette Systemexplantation ist hingegen ausdrücklich obsolet [4]. Die Extraktion des CIED kann transvenös in spezialisierten Zentren sicher durchgeführt werden. Vor Kurzem wurden die Ergebnisse einer deutschen Registerstudie zur transvenösen Sondenextraktion mittels Laser (GALLERY) publiziert, bei der 24 Zentren insgesamt 2524 Patienten eingeschlossen haben. Die Gesamtkomplikationsrate betrug 4,3 %, darunter 2,1 % Major- (lebensbedrohlich, tödlich, bleibende Behinderung) und 2,2 % Minor-Komplikationen. Die verfahrensbedingte Sterblichkeit lag bei 0,55 %. Die Gesamtmortalität im Krankenhaus betrug 3,56 % [5].

## Fazit für die Praxis

- Bei allen Patienten mit CIED und einer nicht hinreichend erklärbaren bzw. rezidivierenden Bakteriämie muss an die Möglichkeit einer CIED-Infektion gedacht werden.
- Bei CIED-Infektion ist immer die komplette Extraktion (Aggregat + Sonden) erforderlich. Alternativ muss eine chronische Suppressionstherapie mit Antibiotika erfolgen.
- Die Extraktion sollte zeitnah nach Diagnosestellung in spezialisierten Zentren erfolgen.
